# Prevention of allergies in childhood – where are we now?

**DOI:** 10.5414/ALX01807E

**Published:** 2017-08-04

**Authors:** B. Ahrens, D. Posa

**Affiliations:** Department of Pediatric Pneumology and Immunology, Charité, Universitätsmedizin, Berlin, Germany

**Keywords:** prevention, food allergies, respiratory allergies, childhood

## Abstract

Allergic diseases represent an increasing health problem for children worldwide. Along with allergic airway diseases, food allergy comes to the fore and herewith closely intertwined the hypothesis that an early allergic sensitization might occur via skin barrier defect(s). The importance of the skin barrier has been documented by several studies meanwhile. Not only genetic studies screen the associations between Filaggrin loss-of-function mutations, atopic dermatitis, allergic sensitization, food allergy and even airway diseases, but also epidemiological studies cast new light on the hypothesis of the atopic march. As another focus in context of the development of an allergic phenotype, the specific microbial exposure with all its diversities has been crystallized as it shapes the immune system in (early) infancy. Studies explored both, the role of human intestinal microbiota as well as the external microbial diversity. Unfortunately suitable markers for atopic predictors are still rare. New studies point out that specific IgE antibodies (e.g., IgE to Phl p 1) in children without allergic symptoms so far, might function as a pre-clinical biomarker, which may help to identify candidates for primary (allergen non-specific) or secondary (allergen-specific) prevention in terms of specific immunoprophylaxis. These manifold research activities document a complex increase in knowledge. Nevertheless new assumptions need to be substantively confirmed in order to finally generate the urgently needed preventive strategies for allergic diseases in childhood.

German version published in Allergologie, Vol. 39, No. 4/2016,pp. 145-159

## Burden, risk and protective factors of childhood allergies 

Data from the worldwide International Study of Asthma and Allergies in Childhood (ISAAC) (meanwhile in Phase III) and also from many other studies, suggest that in less developed countries the prevalence of atopic diseases seems to increase continuously, whereas in affluent societies the asthma-prevalence may have reached a plateau [1, 2, 3, 4]. In Ireland, where in the 1990s the 12-month prevalence of asthma among adolescents was fourth highest in the world [5], a recent report shows that the prevalence of asthma of Irish school children aged 6 – 9 years remained stable at a high level (with 23,5%) between 2002 – 2007 [5]; by contrast, both, the prevalence of allergic rhino-conjunctivitis (AR) (from 7.6% to 10.6%) and that of atopic dermatitis (AD) (from 8.9 to 13.5%) continued to increase. Similarly, the German Health Interview and Examination Survey for Children and Adolescents (KiGGS, age 0 – 17 years), has revealed comparable prevalence rates for the life-time prevalence of AD (13,2%) and for AR (10.7%), while the prevalence for asthma was stable at a much lower level (4.7%) [6]. In South America, the comparison of ISAAC phase I and phase III studies has shown little differences in the prevalence distribution of asthma and AR, although in general the prevalences of asthma vary widely within and between South American countries [8]. Current AR in 6- to 7-year-old old children ranged from 5.5% in Rosario (Argentina) to 21.2% in Caracas (Venezuela). Likewise, current asthma in 6- to 7-year-old children ranged from 10% in Bogota (Colombia) to 31.2% in Sao Paolo West (Brazil). By contrast, in Asia, the prevalence rates of asthma and other allergies remained stable at a relatively low level [9]. 

An increase in food allergies (FA) has been observed for several decades at most. This increase has been called the “second wave of the allergy epidemic“ [10, 11]; however, reliable data are still rare [11, 12, 13]. Not least because only few epidemiologic studies utilize the gold standard of FA diagnosis, the DBPCFC (double-blind placebo-controlled food challenge) [11]. A systematic review and meta-analysis about the epidemiology of FA in Europe calculated the pooled life-time and point prevalence of self-reported FA at 17.3% (95% CI: 17.0–17.6) and 5.9% (95% CI: 5.7 – 6.1), respectively, but challenge-verified FA at 0.9% (95% CI: 0.8 – 1.1), with a north-south prevalence divide within Europe. Similarly, a population-based Australian cohort showed the estimated prevalence rate in 1-year-old infants to be 3.0% for peanut allergy, 8.9 % for raw egg; and 0.8% for sesame [14]. In the United Kingdom, 2% of 8-year-olds were estimated to have peanut allergy [15]. In contrast to these oral food challenge results, data from Canada suggest an overall rate of 8% for self-reported FA (6.7% in the general population), 7.1% of whom were children (most common allergens: cow’s milk (2.2%), peanut (1.8%), and tree nuts (1.7%)) [16]. Interestingly, „certain geographic associations“ with FA have been described [17] which are reflected by the prevalence of certain allergens but also by the „pattern of immunologic reactivity to individual allergenic components within the food“ [17]. Fortunately, many children tend to outgrow their FA. Unfortunately, recent data shows that for cow’s milk allergy (CMA) tolerance development not as good as previously reported. They found tolerance rates of 19% by the age of 4 years, 42% by the age 8 years, 64% by the age 12 years, and 79% by the age 16 years. Besides, children with persistent CMA tend to have higher sIgE levels or coexisting asthma or AR [18]. 

The last-mentioned study illustrates the often-observed deep connection between the allergic diseases [18]. From the 807 included children with CMA (median 13 months), 49% had coexisting asthma, 40% had AR, and 71% had AD. The hypothesis of the „atopic march“, has gained new attention, not least because of data dealing with the barrier function. For some time now, and not least in a systematic review and meta-analysis, the strong relation between Filaggrin gene (FLG gene) mutations and atopic eczema has been documented [19]. Likewise, it has been shown, that these mutations increased the risk of asthma – however mainly in individuals with AD. In a recent Australian study, FLG gene mutations adjusted for AD were associated with food sensitization (OR 3.0; 95% CI, 1.0 – 8.7; p = 0.043), which supports the hypothesis that a barrier defect promotes sensitization [20]. However, very recently the longitudinal relationship between three common Filaggrin gene loss-of-function (FLG-LOF) mutations and FA was explored, taking advantage of data of the Isle of Wight birth cohort [21]. These results not only depict the association of FLG-LOF mutations with eczema and food allergen sensitization, but also underline its role in the development and persistence of FA. Several studies have shown that AD is a strong risk factor for IgE-mediated food allergy [22]. In addition, other barrier defects, for instance tight junctions defects (like claudin-1), are being discussed as other/further predisposing genetic factors in AD [23]. Skin barrier defects in general have meanwhile been established in the “list of risk factors” for (food) allergies (atopic march) together with heredity, route and timing of exposure to food allergens (e.g., introduction of solid foods), vitamin D sufficiency, dietary fat, antioxidants, obesity, exposure to infections, and other factors [17]. G. Lack [17] even postulated the „dual-allergen exposure hypothesis for the pathogenesis of FA, implicating, that allergic sensitization results from cutaneous exposure, and tolerance occurs as a result of oral exposure to food“. In this context, peanut allergen has been detected in house dust and bed even when patients were not aware of having eaten peanuts [24]. As infants spend most of their time in bed, their close contact with the allergen (e.g., peanut protein) via the skin could be considered an important risk factor for environmental sensitization – especially in children with skin barrier defects. 

**Figure 1. Figure1:**
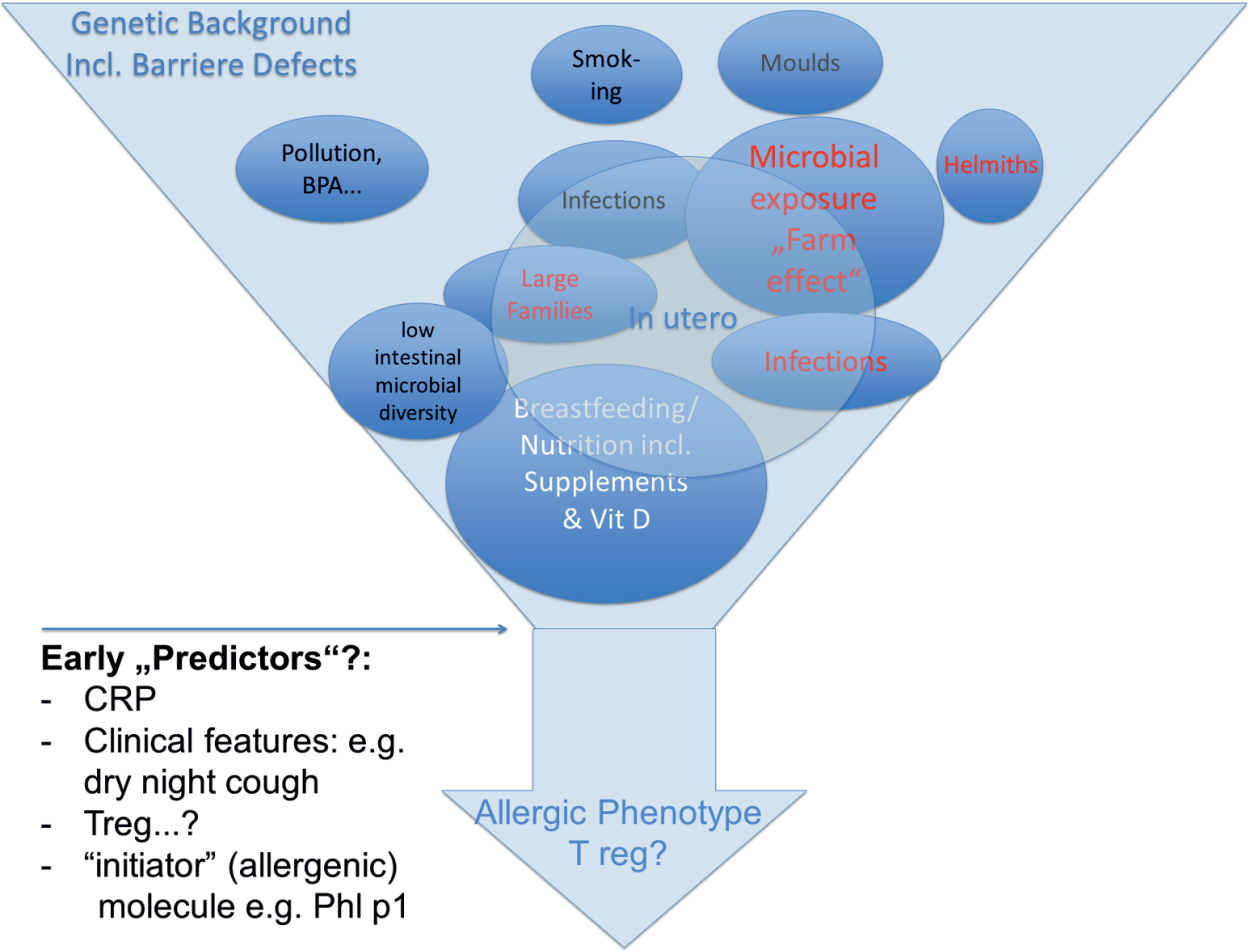
Following risk factors and possible early predictors are discussed in the manuscript.

The hypothesis of the “atopic march” is supported by genetic as well as by epidemiologic observations. Data from a Swedish cohort recently showed that having a FA or being food sensitized is, next to AD or parental allergic diseases or male gender, per se a risk factor for the development of AR in 4.5- and 8-year-old children [25, 26]. [Fig Figure1]

## Microbial exposure and infections 

Since the first verbalization of the “hygiene hypothesis” nearly 25 years ago [27], many supporting environmental factors have been confirmed or even refined. The hypothesis assumes that a genetic predisposition in combination with environmental “hygiene” (less contact to microbial variations or less infections) shape the individual development of allergic diseases because of an aberrant stimulation of the immune system. This hypothesis was first formulated on the basis of the allergy protective influence of a large family size [27, 28]. The GABRIELA study, examining almost 80,000 children aged 6 – 12 years from four central European areas, recently showed that “farm” and “large family size” distinctly reduce the prevalence of hay fever and may therefore underlie different biological mechanisms [29]. The allergy-protective influence of living on a farm and being in contact with a very broad variety of animals has already been reported by several studies. Interestingly, a meta-analysis could examine more than 50 original articles from 39 studies [30] and calculated an approximately 25% lower asthma prevalence in children living on a farm [30]. 

The farming environment seems to influence the development of immunoregulatory components in a significant way. An experimental study showed that isolated-reared piglets had a significantly reduced CD4(+) CD25(+) Foxp3(+) regulatory T-cell population compared to farm-reared littermates [31]. Likewise, a positive correlation and influences of TH17 and Treg cell markers by maternal farm exposure were found in (human) cord blood [32]. Moreover, increased numbers of Treg lymphocytes were found in children regularly drinking farm milk, suggesting that a regulatory phenotype early in life may mediate the allergy-protective effect of farm milk [33]. This result confirms formerly published data showing that microbial exposure, farm milk, and grass components (in cowshed dust) are critical factors of the „farm effect“, as they were repeatedly found inversely associated with asthma and allergy [34]. 

The influence of a high microbial exposure or contact to animals is not invariably positive and it is probably dependent on settings. A recent meta-analysis using 26 publications from 21 birth cohort studies found a favorable effect of exposure to dogs, which decreased the risk of AD in children by nearly 25%, whereas no association was found with exposure to cats [35]. These authors also suggest that these results might be shaped by specific microbial exposure on the immune system in early infancy [35]. The Wheezing Illnesses Study Leidsche Rijn (WHISTLER) investigated the association of exposure to endotoxin (from gram-negative bacteria), house dust mite, and cat allergens (in house dust samples), with neonatal lung function before 2 months of age and respiratory symptoms in the first year of life [36]. In this study, endotoxin in mattress dust was associated with a significant increase in neonatal respiratory compliance but no associations between allergen exposure and neonatal lung function or respiratory symptoms were found, suggesting that environmental exposure to endotoxin (but not to allergen) may influence the development of lung function [36]. Likewise, the overall amount of microbes in house dust samples collected at 2 months of age, was found significantly associated with asthma at 6 years of age [37]. In this study a score of microbial exposure was obtained by summing up indicators for fungi (ergosterol), Gram-positive (muramic acid), and Gram-negative (endotoxin) bacteria, which in the end may predicted asthma better than “single microbial markers independently of microbial diversity and amount of dust” [37] as the diversity score „decreased the risk of wheezing and was significantly (inverted-U shape) associated with sensitization to inhalant allergens“. 

On the other side, indoor fungal diversity (Penicillium, Aspergillus, and Cladosporium) may facilitate exacerbation or even the onset of airway symptoms of asthma as has been shown in a recent meta-analysis [38]. Accordingly, children suffering from asthma may be more susceptible to exacerbations when exposed to outdoor fungal spores [39]. 

Next to “external” microbial diversity, an association between a “more diverse intestinal microbiota early postnatal” with a reduced risk of AD until the age of 12 months in high-risk infants has been observed [40]. Similarly, an association between low intestinal microbial diversity during the first month of life and subsequent AD has been demonstrated [41]. The results of these studies strongly suggest a role of the microbiota in the development of AD. Moreover, the ‘‘beneficial’’ influence of older siblings on the composition of the microbiota suggest that this microbiota may be one of the biological mechanisms underlying the sibling effect as uncovered within a randomized, placebo-controlled trial on the prevention of AD by oral supplementation of a bacterial lysate [42]. 

The overall scenario is even more complex. Indeed, there is an ongoing debate on which infections and vaccinations might have a protective or supporting effect on the development of allergic diseases. Less asthma, AR, and AD (but not FA) were found in 8 year-old children who had been infected by wild-type varicella zoster infection (WTVZV), suggesting that this virus may have suppressed IgE production and allergic sensitization and induced altered leukocyte distributions [43]. By contrast, an ISAAC phase-II study reported that pertussis and measles infections (but not immunization) have been positively associated with asthma, AR, and AD, independently of IgE-mediated mechanisms [44]. Lastly, no evidence on allergic sensitization was detected in the Swedish ALADDIN birth cohort study, in context with exposure to herpes virus infections (EBV, HHV6, HHV7 or CMV), suggesting that these infections might not contribute to allergy protection [45]. 

In parts, data contradicting former observations have been gained with regard to TB disease and bacillus Calmette-Guérin (BCG) vaccination in early life as well as the nexus between helminth infections and allergic diseases, reflecting the complex area of epidemiological and experimental research and the link between allergic inflammation and susceptibility of the influence on the immune system [46, 47, 48]. 

## Tobacco smoke and other environmental factors 

The Tucson Epidemiological Study of Airway Obstructive Disease (TESAOD) showed that exposure to parental tobacco smoke in childhood leads to an increased risk of persistent respiratory symptoms into young adult life [49]. Similarly, children with AA genotype for GSTP1, an isoform of the glutathione S-transferases (GSTs), which detoxify xenobiotics from tobacco smoke in the human lung, may have a higher risk of wheezing in early life, if their mother smokes [50]. Both publications again document the importance of (passive) smoke reduction in the presence of children to reduce their risk for development or worsening of respiratory symptoms. However, a meta-analysis based on the data of 5 European birth cohorts did not find clear evidence of an association between air pollution exposure and development of allergic sensitization in children of up to 10 years of age [51]. 

As rather new environmental toxins, the endocrine-disrupting chemicals phthalate (prenatal exposure) and bisphenol A (BPA) (exposure in early childhood) are reported to be associated with an increased asthma risk in inner-city children [52, 53]. 

## Breast-feeding, maternal and infant diet 

The influence of the maternal diet on the development of allergic diseases in the offspring has been one central research topic of the last years. A recent prospective Finnish birth cohort adds that maternal consumption of leafy vegetables, pome fruits, and chocolate during pregnancy may protect the offspring from the development of wheeze at 5 years of age, while no association between asthma and maternal diet was found [54]. A Cochrane meta-analysis concluded that the allergen-restrictive diet of a pregnant high-risk woman is not likely to reduce the risk for AD in the child, while a special diet during lactation may influence the development of AD, although sufficient data is missing [55]. In contrast, it has been shown, that after maternal consumption of, e.g., peanut, Ara h 6 is measurable in human breast milk as soon as 10 minutes later. Interestingly, when giving this human milk to young mice, these do not get sensitized but develop partial oral tolerance. It is not clear whether there is as a preventive rather than a sensitizing effect when the active allergens are transferred to the children through breast milk [56]. 

The impact of breastfeeding per se on asthma and allergic sensitization is an ongoing debate. The database of two cohort studies (the CAPStudy, Australia, and the BAMSE cohort, Sweden), which initially reported different findings, were combined, and definitions for outcome parameters were harmonized. The “new” interpretation of data suggests that breastfeeding reduces the risk of asthma up to 8 years of age but seems a risk factor for sensitization to different food allergens [57]. The authors withhold a recommendation for or against breastfeeding, but underline the importance of the ” harmonization of features of study design” [57]. By contrast, two other studies emphasize the potential protective effect of breastfeeding against allergies, as the specific neonatal innate immune function was associated with breastfeeding [58] and its duration was associated with the levels of IL-10 and RANTES secreted by the upper respiratory tract of preterm children 1 year after birth [59, 60, 61]. 

The role of diet in early life is also being intensively investigated. Perhaps the most important finding is that a restricted food pattern during the first year of life might increase the risk of asthma and allergies in childhood [62], whereas an “increased diversity of food within the first year of life seems to have a protective effect on asthma, food allergy, and food sensitization and is associated with increased expression of a marker for regulatory T cells” [63]; comparable results have been reported from the Prevalence of Infant Food Allergy (PIFA) study [64]. In a review and meta-analysis, Garcia-Marcos et al. [65] deduced a trend to a protective effect of a Mediterranean diet against the occurrence of asthma in childhood. Fish seems to play a role, as in a recent prospective, longitudinal cohort study from Sweden a protective effect of early introduction (before the age of 9 months) of fish into the child’s diet with regard to asthma prevention has again been confirmed. A remaining protective effect has even been described up to an age of 8 years [66]. 

Generally speaking, the trend to recommend a balanced and nutritionally sufficient diet for pregnant or lactating mothers as well as for infants and children, while avoiding obesity, goes on [62, 63, 64, 65, 67, 68]. Delaying the introduction of solid food did not show preventive effects in children with high or normal risk for FA [68]. Primary and secondary prevention studies are running in different countries (see Prevention). Their outcome will contribute to the present recommendations [68]. 

The evidence for preventive effects of the intake of supplements by pregnant or breastfeeding women is so far considered weak [68]. For a considerable time, the role of (low) Vitamin D in allergic diseases has been vividly discussed [69]. Vitamin D is considered to play a key role in innate and adaptive immunity by stimulation of toll-like receptors, by increasing pro-inflammatory cytokine production, and possibly by enhancing T-helper type 2 responses [69]. However, definitions of the optimum serum level (with regard to „global health” as well as to the “immune system function and not only for its effects on muscle and skeletal functions”), and the levels representing vitamin D deficiency or insufficiency are not clear [70]. Low serum 25-hydroxyvitamin D levels have been associated with a higher risk of upper and lower respiratory tract infections in children [69, 70]. The suggestion to perform vitamin D screening in uncontrolled asthmatics has (again) been underlined by a recent study among Israeli adults, where low vitamin D levels were associated with asthma exacerbations [71]. Based on observations of associations between childhood asthma, fetal lung and/or immune development, and maternal vitamin D intake, a mouse model was used, where adult offspring of vitamin D(3)-repleted and vitamin D(3)-deficient colonies of BALB/c mice showed enhanced allergen-induced lymphocyte responses. This might be interpreted to mean that vitamin D(3) deficiency modulates the capacity of lymphocytes to respond to allergens [72]. Nevertheless, although there is increasing data supporting a role of vitamin D in the immune system and allergic diseases, the meaning of its influence has still not been fully explored. 

Our extraction of data give an example of the great achievements that have been made [73]: starting from the observation of a protective effect of living on a farm, where a high microbial diversity is present, followed by the stepwise elaboration of the importance and immune-modulating power of certain parameters [74]. Nevertheless, the identification of the precise underlying mechanisms still remains to be completed, but the manifold gain of knowledge about different risk factors of allergic diseases due to research activities, contributes to a more comprehensive view of the complex interplay between genetics and environment, including the influence of an “allergy-correct diet”. The complexity and inconsistency of the databases require much more effort to finally develop prevention strategies for the individual patient. 

## Prevention of childhood allergies 

A crucial point for prevention is

- who should start preventive strategies? and
- when should be they started?

To answer these questions, suitable predictors are of great value [75]. Some studies will be presented that were carried out to identify such risk markers. For example, a current study demonstrated that, in their collective, elevated maternal concentrations of C-reactive proteins (CRP) during pregnancy were associated with an increased eczema risk in the child and that increased CRP values in cord blood were related to an increased risk of wheezing and airway infections during the child’s first years of life [76]. In contrast, two publications from the PASTURE study group, postulated, that a low-grade inflammation due to infectious agents in early childhood might protect from allergic diseases later in life. They observed that elevated levels of high-sensitivity CRP at the age of 1 year were associated with decreased allergic sensitization [77], whereas in 4.5-year-old children their observations have been less strong [78]. 

The Pollution and Asthma Risk: an Infant Study (PARIS) birth cohort suggests that persistent/late night cough (even in absence of wheeze) may point to allergy in preschool children [79]. 

In the context of the phenomenon of “molecular spreading” (specific IgE response starts with an “initiator” (allergenic) molecule from monosensitization to oligomolecular and finally to polymolecular sensitization), the idea for an earlier immunological intervention came up [80]. Phleum pratense (Phl p1) has been documented as an „initiator“ molecule in children without clinically manifest symptoms of AR so far. Nevertheless, a pre-school child with IgE to grass pollen is likely to develop subsequent AR, especially with a positive family history for allergies. Therefore, specific pre-clinical immunoprophylaxis, even before symptoms are present, has been suggested. 

Concerning the prediction of food allergies, other study groups concentrated on IgA and IgG antibodies against β-lactoglobulin in cow’s milk and gliadin in wheat. As documented in the PASTURE study, these might function as markers of gut permeability and inflammation, both of which influence development of mucosal tolerance. The authors found that at age 1 an increase of these markers was associated with IgE sensitization at age 6 [81]. The authors concluded that enhanced response due to mucosal aberrancies, e.g., altered microbiota and/or increased gut permeability, could later be seen as sensitization to allergens [81]. In another study, low β-lactoglobulin-specific serum IgG4 levels in the serum of children with eczema and allergy to cow’s milk were lower than in children with eczema who did not have an allergy to cow’s milk [82]. 

## Primary prevention 

All of these and other factors of allergic pathogenesis can be verified or rebutted in well-designed interventional studies. As an example, interventional studies on the primary prevention of asthma in high-risk children showed that the strict avoidance of certain airborne and food allergens (e.g., dog, cat, house dust, or cow’s milk) during early childhood does not influence the development of childhood allergic asthma, although this had been suggested in observational studies [83, 84, 85]. 

Current infant feeding guidelines in many affluent countries consider hydrolyzed formula as the primary prevention therapy for allergic disease. Nevertheless, the benefit of partially (PHF) and extensively hydrolyzed cow’s milk formula (EHF) has been questioned not least by new evidence from a large interventional trial in high-risk infants (the Melbourne Atopic Cohort Study) [86, 88]. Likewise, new data from the GINI study show little evidence „of an ongoing preventive effect between the ages of 7 and 10 years” [86, 87, 89]. Therefore, adversaries state that it may currently be premature to recommend hydrolyzed formula for the prevention of allergic disease including eczema [86]. On the other hand, promoters believe that hydrolysates with proven efficacy should be employed and recommended for high-risk children who cannot exclusive be breastfed in their first 4 – 6 months of life [90]. This debate has not yet been completed and, as many times suggested, more studies are needed [91]. 

Regarding the time point of the introduction of solid foods in the child’s diet, ongoing interventions studies try to find an answer (please see above) [92]. 

Another hotly debated topic is the use of probiotics in the primary prevention of allergic diseases. Abrahamsson et al. [93] recently published a 7-year follow-up of a randomized, placebo-controlled trial with perinatal and early-childhood oral supplementation with L. reuteri ATCC 55730. Although an effect of L. reuteri on sensitization and IgE-associated eczema in infancy was observed, respiratory allergic diseases at school age were not prevented [93]. Two reviews and a meta-analysis [94, 95] regarding the effect of probiotics during pregnancy or infancy for the prevention of atopic diseases came to a comparable result. One included 20 randomized trials enrolling a total of 4,866 infants and showed an extreme heterogeneity in therapeutic approaches: probiotic supplements had been given orally by capsules, oil droplets, suspensions in water, milk, or infant formula; exclusively prenatal maternal or exclusively postnatal or combined; including various probiotic organisms: Bifidobacterium species (B bifidum, B longum, two strains of B breve, and four strains of B lactis), Lactobacillus species (L acidophilus, L casei, L lactis, L reuteri, strains of L paracasei, strains of L rhamnosus); exclusively or combined with prebiotics. The authors finally conclude that they did not find any evidence to support a „protective association between perinatal administration of probiotics, and doctor diagnosed asthma or childhood wheeze.“ Furthermore, they point out that there is currently insufficient evidence to recommend probiotics for the primary prevention of these disorders, and further research is warranted [94]. Another meta-analysis included 25 studies of 20 cohorts with a total of 4,031 participants [95]. Applications and choice of probiotic strains were, in general, similar to the above-mentioned meta-analysis. The authors concluded that probiotics in early life may reduce total IgE and protect against atopic sensitization but that they do not protect against asthma and wheeze [95]. 

Vitamin D is also considered to have a protective effect on the genesis of atopy. Surprisingly, a double-blind, placebo-controlled interventional study from Japan showed that maternal Vitamin D supplementation (n = 82) did not have a positive effect on the development of childhood eczema at the age of 3 months (primary outcome). Instead, the risk of having developed food allergy by the age of 2 years (secondary outcome), seems to have been increased [96]. Due to the high number of dropouts, among other things, the authors stated that these observations would have to be confirmed in further studies [96]. 

Studies on the (stabilization of the) skin barrier follow an entirely different approach of primary prevention. It has been postulated that early interventions, like restoring the skin barrier, prevent AD and could influence associated comorbidities, llike AR or allergic asthma [97, 98, 99]. First pilot studies with emollient therapy are very promising [100]. Moreover, improved emollient use reduces severity of AD in children and has been shown to be cost-neutral [101]. There has been no data so far about the impact of improved emollient therapy on the sensitization status or even on its influence on the atopic march. 

## Secondary prevention 

The first publication of a randomized controlled trial to investigate whether early introduction of egg reduces the risk of egg allergy in infants with a history of eczema shows that an induction of immune tolerance might be possible by early regular oral exposure to egg from 4 months of age in infants with moderate-to-severe eczema [102]. Nevertheless, the authors emphasize that these kind of studies bare risks, as they deal with children at high risk for food allergic reactions, and children might be sensitized even before they start eating solid food. 

Interestingly, another Australian study showed that less children who had reacted allergic to baked egg allergy at the age of 1 year lost their allergy to raw egg as compared to children who had tolerated baked egg at the age of 1 year (13% and 56%, respectively; adjusted odds ratio, 5.27; 95% CI, 1.36-20.50; p = 0,02). These new data (baked egg tolerant vs. allergic) suggests that with regard to tolerance development a subphenotype “baked egg” exists [103]. 

Other ongoing intervention studies (RCTs Randomized Controlled Trials) dealing with tolerance development due to early and frequent food allergen consumption are: e.g., the BEAT “Beating Egg Allergy” Study from Australia (n = 600) or the PEAP “Peanut Allergy Prevention” study from Germany (n= 460), and others [92]. 

The results of the „Learning Early About Peanut Allergy study, LEAP“ published early this year came like a thunderbolt [104]. This prospective, randomized intervention trial involves 640 high-risk children who were enrolled at age 4 to 10 months and assigned to a peanut consumption or a peanut avoidance group up to the age of 5 years. The results indicate that an early introduction of peanut indeed could prevent peanut allergy. Even more, the early and frequent „consumption was effective not only in high-risk infants who showed no indication of peanut sensitivity at study entry (primary prevention) but also in infants who had slight peanut sensitivity (secondary prevention)“ [105]. Although these results are compelling, there are still many open questions, for instance regarding dosage and duration of peanut intake. Likewise, it has to be questioned whether this observation is transferable to other foods [105]; especially as there are discussions that every food allergen may have its own „window of opportunity“ for development of tolerance or food allergy [106]. Therefore, hopefully the results of other, ongoing intervention studies will help to answer at least parts of so far open questions [92]. 

Finally, besides all the efforts and research activities, the child itself should not be neglected. Interestingly, farm children report a higher health-related quality of life, probably because of less atopic diseases than non-farm children [107]. By implication this underlines the need for prevention strategies for children with chronic atopic diseases, but also their susceptibility should be considered and the wish for educational strategies next to medical support [108, 109]. 

## Conclusion 

Many factors, which are discussed above „fail“ or „pass“ when looking at results of large epidemiological studies. For example, to determine early-life predictors of asthma incidence up to age 20, data from the Multicenter Allergy Study was used and evaluated by “time-to-event analysis” [110]. The results show that:

- asthma incidence was lower in vaccinated participants;
- asthma incidence was higher in children of parents with: - positive history of allergies, - increased cord blood IgE levels, - who started day-care before an age of 18 months or after an age of 3 years, - who had mothers who smoked during pregnancy, - who had poor parents or grandparents.

No association was found:

- regarding aspects of diet (“breastfeeding, weaning, diet in pregnancy, and parental food hypersensitivity),
- mode of delivery,
- pet ownership (cat or dog),
- age of mother,
- presence of older siblings,
- and tobacco smoke exposure”.

The results make clear, that any observations, even done in prospective cohort studies, are not as good as randomized controlled, prospective (intervention) trials with a preferably precise hypothesis and target group. Even these results need to be confirmed and have to be consistent. 

Data of many ongoing studies regarding dietary aspects of mother and child, regarding supplements (e.g., vitamin D), intervention and analysis of the human microbiome, and dealing with skin barrier defects will be included in the current S3 guidelines on allergy prevention and will help to develop better strategies for prevention ((http://www.awmf.org/uploads/tx_szleitlinien/061-016l_S3_Allergiepr%C3%A4vention_2014-07.pdf).
